# The Health*e*Steps™ lifestyle prescription program to improve physical activity and modifiable risk factors for chronic disease: a pragmatic randomized controlled trial

**DOI:** 10.1186/s12889-019-7141-2

**Published:** 2019-06-28

**Authors:** D. P. Gill, W. Blunt, N. C. Boa Sorte Silva, C. Stiller-Moldovan, G. Y. Zou, R. J. Petrella

**Affiliations:** 1 Centre for Studies in Family Medicine – Department of Family Medicine, Schulich School of Medicine and Dentistry, Western University, 1465 Richmond St., London, ON N6G 2M1 Canada; 2 School of Health Studies, Faculty of Health Sciences, Western University, London, ON Canada; 3 School of Kinesiology, Faculty of Health Sciences, Western University, London, ON Canada; 4 Department of Epidemiology and Biostatistics, Schulich School of Medicine and Dentistry, Western University, London, ON Canada; 5 Robarts Clinical Trials Inc., London, ON Canada; 6 Lawson Health Research Institute, London, ON Canada

**Keywords:** Lifestyle intervention, Pragmatic randomized controlled trial, Physical activity, Healthy eating, Health behaviours, Chronic disease, *e*Health

## Abstract

**Background:**

Our objective was to determine the influence of the Health*e*Steps™ lifestyle prescription program on physical activity and modifiable risk factors for chronic disease in individuals at risk.

**Methods:**

One hundred eighteen participants were recruited from 5 sites in Southwestern Ontario, Canada and randomized to either the intervention (Health*e*Steps™ program, *n* = 59) or a wait-list control group (*n* = 59). The study comprised three phases: an *Active Phase* (0 to 6 months) consisted of bi-monthly in-person lifestyle coaching with access to a suite of *e*Health technology supports (Heathesteps app, telephone coaching and a private Health*e*Steps™ social network) followed by a *Minimally-Supported Phase I* (6 to 12 months), in which in-person coaching was removed, but participants still had access to the full suite of *e*Health technology supports. In the final stage, *Minimally-Supported Phase II* (12 to 18 months), access to the *e*Health technology supports was restricted to the Health*e*Steps™ app. Assessments were conducted at baseline, 6, 12 and 18 months. The study primary outcome was the 6-month change in average number of steps per day. Secondary outcomes included: self-reported physical activity and sedentary time; self-reported eating habits; weight and body composition measures; blood pressure and health-related quality of life. Data from all participants were analyzed using an intent-to-treat approach. We applied mixed effects models for repeated measurements and adjusted for age, sex, and site in the statistical analyses.

**Results:**

Participants in Health*e*Steps™ increased step counts (between-group [95% confidence interval]: 3132 [1969 to 4294], *p* < 0.001), decreased their sitting time (− 0.08 [− 0.16 to − 0.006], *p* = 0.03), and improved their overall healthful eating (− 1.5 [− 2.42 to − 0.58], *p* = 0.002) to a greater extent compared to control at 6 months. Furthermore, exploratory results showed that these individuals maintained these outcomes 12 months later, after a minimally-supported phase; and retained improvements in sedentary time and improved healthful eating after 18 months. No differences in self-reported physical activity, health-related quality of life, weight, waist circumference or blood pressure were observed between groups at 6 months.

**Conclusions:**

Our findings suggest that Health*e*Steps™ is effective at increasing physical activity (i.e., step counts per day), decreasing weekday sitting time, and improving healthful eating in adults at increased risk for chronic disease after 6 months; however, we did not see change in other risk factors. Nonetheless, the maintenance of these behaviours with minimal support after 12 and even 18 months indicates the promise of Health*e*Steps™ for long-term sustainability.

**Trial registration:**

The trial was registered on April 6, 2015 with ClinicalTrials.gov (identifier: NCT02413385).

**Electronic supplementary material:**

The online version of this article (10.1186/s12889-019-7141-2) contains supplementary material, which is available to authorized users.

## Background

Chronic disease continues to be the leading cause of premature death throughout the world accounting for 68% of all deaths in 2012. Approximately 30% of Canadian adults are living with one or more of the five main chronic diseases such as cancer, diabetes, cardiovascular diseases, chronic respiratory diseases, and mood and anxiety disorders [1, 2]. This rate is only expected to grow as the Canadian population ages and continues to engage in risky health behaviours [3]. In 2015, 84.7% of Canadians aged 20 and older reported having one or more of the main modifiable risk factors for chronic disease, which include being sedentary, physically inactive, smoking, having poor eating habits, and consuming alcohol in excess [2]. This rise in chronic disease carries a significant cost to the Canadian health care system with estimates suggesting treatment and lost productivity for those with chronic diseases costs $190 billion annually [4].

Many current and future cases of chronic disease can be prevented and managed through healthy lifestyle changes such as meeting the recommendations for physical activity, decreasing sedentary behaviour, and eating a healthy diet [5]. Physical inactivity is of particular concern in Canada as only 17.5% of adults meet the recommendations for physical activity [2]. Even small increases in levels of physical activity can have a significant impact on reducing chronic disease risk [6]. Studies exploring walking have found that those who walk at least 2 h per week have a 39% lower all-cause mortality rate than those who walked less than 2 h a week [7]. Combining physical activity with another healthy lifestyle behaviour, such as a healthy diet, can reduce chronic disease risk even further and tends to be a stronger approach to preventing and managing chronic disease as most individuals engage in more than one risk factor at a time (i.e., sedentary behaviour and poor diet) [8].

Health*e*Steps™ is a chronic disease prevention and management program developed from an extensive research base analyzing physical activity counselling practices of primary care clinicians [9, 10], lifestyle interventions to improve cardiovascular health [11, 12], and use of health technology to improve lifestyles of chronic disease patients [13–15]. The Health*e*Steps™ program pulls aspects from the Social Cognitive Theory of self-regulation [16], utilizing self-monitoring, and goal setting. Health*e*Steps™ coaches are trained using the Co-Active coaching model, which acknowledges the participant as the expert in their life and health [17]. Coaches collaborate with the participant to assist with setting goals using standardized readily available healthy living resources and discuss strategies to achieve those goals that are tailored to the participants local environment. The program utilizes self-monitoring beyond the in-person coaching sessions, whereby participants are provided with paper tracking forms to monitor their progress with their goals each day in between coaching sessions.

Participants met one-on-one with a trained Health*e*Steps™ coach every other month for 6 months. During these coaching sessions, participants worked with their coach to set prescriptions (goals) for physical activity (steps/day) and exercise (moderate to vigorous activity) guided by the *Canadian Physical Activity Guidelines for Adults 18–64 years* [18], and healthy eating based on the recommendations for healthy eating provided from *Eating Healthy with Canada’s Food Guide* [19]. These prescriptions are set using S.M.A.R.T. goal setting principles to ensure they are specific, measureable, attainable, realistic, and timely [20]. The physical activity prescription was focused on reducing sedentary behaviour (i.e., by increasing steps/day and reducing sitting time), while the exercise prescription was focused on improving cardiovascular functioning. The program is also supported by innovative technology resources: an online social network; phone coaching; and the Health*e*Steps™ smartphone app.

The primary aim of this study was to determine effectiveness of the Health*e*Steps™ program in increasing physical activity levels (i.e., average number of steps per day) in individuals at-risk for chronic disease after 6 months. Secondary aims included examining: 1) the impact of Health*e*Steps™ on other health behaviours (i.e., sedentary time, healthy eating) and health indicators (i.e., health-related quality of life, weight, body composition, and cardiometabolic measures); and 2) the maintenance of any positive changes in health behaviours or health indicators after 12 and 18 months (i.e., minimally-supported phases I and II, respectively).

## Methods

### Trial design

The full protocol of the study has been published elsewhere [21]. In brief, we conducted a randomized controlled trial in Southwestern Ontario, Canada where we recruited 118 participants at risk or diagnosed with a chronic disease from five primary care/health care services sites in urban (three sites in London Ontario) and rural (one in Forest, Ontario, and another in Tillsonburg, Ontario) settings. Participants were recruited through primary care referrals from health care providers at the sites, or through program advertisements at these sites. Participants were individually randomized (1:1) to one of two groups: intervention (Health*e*Steps™ program) or comparator (wait-list control). Follow-up occurred at 6 months (both groups), 12 months and 18 months (intervention group only). The Western University Health Sciences Research Ethics Board approved the study and all participants provided written informed consent. The trial was registered on April 6, 2015 with ClinicalTrials.gov (identifier: NCT02413385).

### Participants & setting

Participants were recruited through poster advertisements, word of mouth, health care provider referrals, staff e-mails, and in-person recruitment booths set up at the participating sites. Eligible participants were individuals between 18 and 85 years of age with one or more self-reported or measured risk factors for chronic disease including: body-mass index (BMI) greater than 25 kg/m^2^; less than 150 min of exercise per week; 3 or more hours sitting per day; consuming less than 8 fruit and vegetable servings per day; diagnosis of type 2 diabetes. Clearance to participate also required completion of the Physical Activity Readiness-Questionnaire (PAR-Q) [22] or approval from a health care provider. Participants were excluded if they could not comprehend the letter of information and consent form.

### Intervention group: Health*e*Steps™ program

#### Active phase (month 0–6)

Throughout the active phase, participants met with a trained Health*e*Steps™ coach every other month (months 0, 2, 4, and 6) to set their exercise (moderate to vigorous intensity), physical activity (steps/day) and healthy eating prescriptions and discuss strategies to achieve their goals. Specifically, sessions were personalized to the participant focusing on setting S.M.A.R.T. (specific, measurable, attainable, realistic, and timely) goals. For the exercise prescription, participants completed a validated Step and Exercise Prescription (STEP Test™ [23, 24]) providing a personalized target heart rate to measure and assist participants meeting their personal recommendations for moderate to vigorous activity. Coaches also discussed strategies with participants on how to increase the amount of time that they spent exercising at their target heart rate (i.e., encouraging participants to slowly increase time spent during exercise at their target heart rate). For the physical activity prescription, participants used a pedometer to record their average daily step count for 1 week (baseline). A paper chart was used to guide participants to incrementally increase their step count up to 10,000 steps per day (See Additional file 1: Table S1 for step count guide). For aiding in further reducing sedentary behaviour, participants were instructed to reduce their sitting time in addition to increasing their step count daily. Lastly, participants were asked about their diet from the previous week in order to determine their baseline eating habits. From this, a heathy eating prescription was planned so that the participant would increase (or decrease) their intake of fruits and vegetables, fats, carbohydrates and protein until they met the recommendations set out by Health Canada through *Eating Well with Canada’s Food Guide* [19]. Coaches for the program had a background in health promotion and previous experience delivering healthy lifestyle programs. Coaches were trained in the program protocol in-person by members of the research team prior to program delivery and were advised to work within participants’ abilities and to modify activities/goals based on pre-existing medical conditions such as musculoskeletal conditions (e.g., back or joint pain) and any new injuries.

Participants had access to our free customized health technology (eHealth) tools and resources including: 1) phone coaching through a third-party organization trained in the Health*e*Steps™ protocol, 2) online Health*e*Steps™ social network to connect with other participants and their coach, 3) Health*e*Steps™ smartphone app with a virtual coach and step counter, 4) Health*e*Steps™ website (healthesteps.ca).

Attendance at each of the four in-person coaching sessions was tracked. Participants were classified as a *program completer* if they attended at least two of the four program sessions, including at least one of the final two sessions (i.e., sessions three or four). Sessions were conducted at the clinic sites and lasted 30–40 min.

#### Minimally supported phase I (month 7–12)

Although in-person coaching was removed, participants during this phase continued to have access to the eHealth tools and resources. Participants were encouraged to continue to make healthy lifestyle changes as prescribed at their last in-person session.

#### Minimally supported phase II (months 13–18)

The online Health*e*Steps™ social network, and phone coaching supports were removed but participants still had access to the Health*e*Steps™ smartphone app and Health*e*Steps™ website.

No set protocol for using Phase I or II minimally supported eHealth tools and resources was provided; participants were encouraged to use the supports as needed. Supports were slowly removed from participants in order to observe the maintenance of behaviour changes using only phone-based and technology resources (i.e., remote support tools).

### Comparator group: wait-list control

Participants were encouraged to continue with their usual activities without restriction and provided publicly available resources related to healthy lifestyles. Participants were only contacted to attend assessments at 6 months and were invited to complete the full 6-month program after the follow-up assessments occurred.

### Measurement

Outcome measures and measurement protocols are described in detail elsewhere [21]. The planned primary outcome for this trial was the difference between groups in the average number of steps per day from baseline to 6 months using the Yamax Digiwalker SW-200 pedometers. Participants self-reported their step counts on a paper log over a 7-day tracking period [25]. We also examined long-term behaviour maintenance within the intervention group by examining within group changes from baseline to 12 and 18 months. Secondary outcomes included:

1. *Self-Reported Physical Activity and Sedentary Time*
  Total physical activity (Metabolic Equivalent (MET)-minutes/week) measured using the International Physical Activity Questionnaire (IPAQ) Short Form [26]
  Time spent in sedentary activity (minutes spent sitting on a typical week day) also measured with the IPAQ
2. *Self-Reported Eating*
  Healthful eating score measured using Starting the Conversation questionnaire [27]
  Fatty food score measured using a modified version of the Dietary Instrument for Nutrition Education (DINE) [28] and following scoring outlined by Hunt and colleagues [29]
  Fruit and vegetable consumption measured using a modified version of the DINE
  Sugary food consumption measured using a modified version of the DINE
3. *Health-Related Quality of Life*
  Self-rated health measured using the EuroQol Visual Analogue Scale (EQ-VAS) [30, 31].
4. *Weight and Body Composition Measures*
  Weight (using Tanita HD 351 Digital Weight Scale; kg)
  Body Mass Index (BMI, calculated from weight and height in kg/m^2^)
  Waist circumference (cm) [32]
5. *Cardiometabolic Measures*
  Resting systolic blood pressure (BP) and diastolic BP (using BP Tru BPM-100; mmHg).

### Adverse events

Any injuries, newly diagnosed health conditions, surgeries, or increases in medication that occurred during the study, regardless of whether it was related to participation in the study, were reported as adverse events. Events that required hospitalization, prolonged medical attention, were immediately life threatening or fatal, were considered serious adverse events. At each coaching session, participants were asked about any adverse events and serious adverse events that may have occurred between sessions and whether it was related to their participation in the study. Coaches recorded these in an adverse event log and participants were asked about the status of any previous adverse events at subsequent coaching and measurement sessions.

### Randomization, allocation, blinding

The randomization sequence was generated using SAS software version 9.4 (SAS Institute Inc., Cary, NC). Randomization was stratified by site (1:1 allocation; block size: 4) and concealed using sequentially numbered and sealed opaque envelopes. Following baseline, an individual from the research team who was not involved in generating the sequence or preparing the envelopes, enrolled participants and assigned them to one of the two groups. Following allocation, all participants (intervention and comparator groups) were provided with copies of *Eating Well with Canada’s Food Guide* [19] and the Canadian Physical Activity Guidelines for Adults [18]. It was not possible to blind participants or coaches in this study.

### Sample size

Informed by literature [33] and as outlined in our previous publication [21], this study was powered to detect an effect size of 0.65 (a moderate to large effect size) for our planned primary outcome (difference between groups in mean change in average steps/day at 6 months). Assuming an effect size of 0.65, 80% power, a 0.05 two-sided significance level, and a conservative 30% loss to follow-up estimate, we determined that 55 participants in each arm (110 total) were required [34]. Since we had five sites who expressed interest in taking part in this study, we proposed that each site needed to enroll 22 participants (i.e., 11 participants per group per site).

### Statistical analysis

We analyzed data based on an intent-to-treat approach and thus, included all participants with at least valid baseline data according to group allocation. In order for step count data to be considered valid and therefore included in the analysis, participants had to have complete step count data for at least three of the 7 days within the tracking period [35]. We examined differences between groups in mean change in average steps/day at 6 months using a linear mixed effects model for repeated measurements and adjusted for age, sex, and site; this model also provides estimates of within group change from baseline to 6 months. We retained the baseline value as part of the outcome and constrained group means as equal because of randomization (i.e., no group term [36]). The model included terms for time (0, 6, 12, and 18 months), group (Intervention, Comparator) × time, age, sex, and site (London 1, London 2, London 3, Forest, Tillsonburg). This approach is equivalent to an analysis of covariance approach, but has the advantage of including subjects with missing follow-up data [37]. We ran an additional linear mixed effects model for repeated measurements adjusted for age, sex, and site within the intervention group only to obtain exploratory estimates for mean change from baseline to both 12 and 18 months.

We followed the same modeling approach for all continuous outcomes. Residuals from models were examined and subjected to assumptions checks. Due to non-normality, transformations were required for two of the secondary outcomes examined; a square-root transformation was applied to total physical activity and a log transformation was applied to sitting time. Accordingly, results for total physical activity are presented on the square-root scale and results from sitting time are presented on the log scale. We used generalized linear mixed effects models (log-linear modified Poisson models) for repeated measures adjusted for age, sex and site for the two categorical-level (binary) outcome variables (fruit/vegetable consumption and sugary foods consumption) and followed the same modeling approach as done with the continuous outcome variables.

To address participant dropout at 6 months, we compared important baseline characteristics of participants who attended 6-month measurement sessions versus those who did not attend 6-month measurement sessions using independent samples t-tests for continuous variables and either Pearson’s chi-square tests or Fisher exact tests for categorical variables. Interpretation of study results is primarily based on estimation and associated 95% confidence intervals. Analyses were performed using SAS version 9.4 (SAS Statistical Analysis Software).

## Results

### Study recruitment

The study occurred between May 2015 and December 2016. Of the participants who were eligible and enrolled in the study: 51 (45%) heard about the study from posters or handouts; 28 (25%) received an email from the study site advertising the project; 15 (13%) found out about the study from an in-person study recruiter (either from the Central Research Team or the site staff); 12 (11%) were referred by their health care provider (HCP) and/or HCP team; 6 (5%) heard about the study through word of mouth; and 1 (1%) heard about the study through other unspecified methods. An additional 5 individuals did not specify how they heard about the study.

### Study flow & participant retention in the trial

**Fig. 1:**
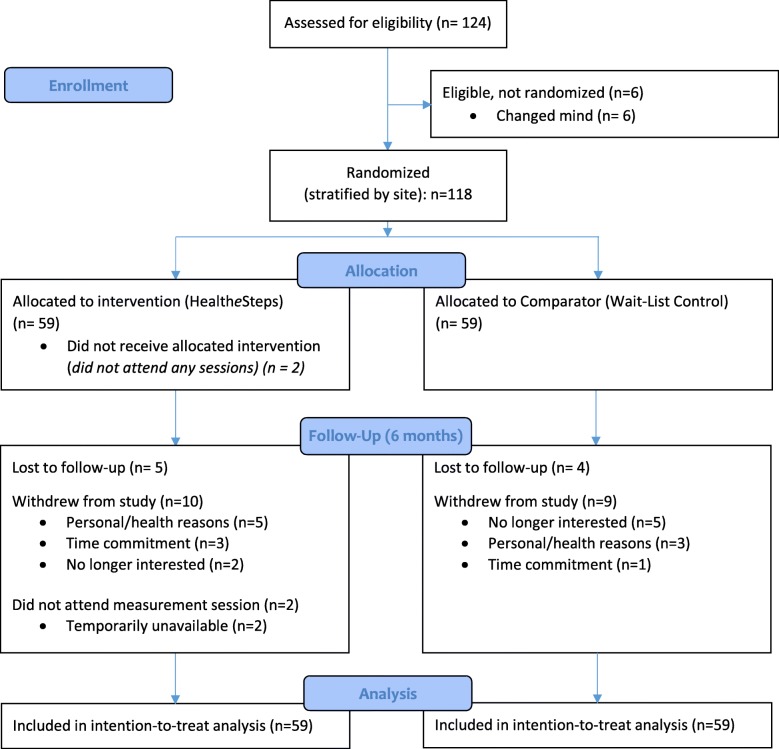
Study Flow Diagram to 6-Month Follow-up (Final Measurement Point within the Comparator Group)

In total, there were 124 individuals across the 5 sites who expressed interest in the study and underwent assessment for eligibility. Due to the pragmatic nature of this study, inclusion criteria were broad and accordingly, no participants were excluded due to ineligibility. Figure 1 outlines the study flow to 6 months, corresponding to the time point of the primary outcome. Of the 124 individuals who were screened, 6 chose not to proceed any further with the study and a total of 118 individuals completed baseline assessments and then were randomly allocated to either intervention (*n* = 59) or comparator (*n* = 59). The breakdown of participants by site was as follows: London site 1 (*n* = 26 [22%]), London site 2 (*n* = 28 [24%]), London site 3 (*n* = 11 [9%]), Forest (*n* = 22 [19%]), and Tillsonburg (*n* = 31 [26%]).

The number of participants who were lost to follow-up or withdrew from the study after 6 months (following the active phase) was similar between the two groups. Specifically, by 6 months, 9 participants were lost to follow-up (5 in the intervention and 4 in the wait-list control group). Another 19 participants dropped out of the study by the end of the active phase (10 in the intervention and 9 in the wait-list control group). An additional 2 participants in the intervention were temporarily unavailable to attend a measurement session at 6 months but remained in the study.

When we compared the 30 participants who did not attend the 6-month measurement sessions with the 88 participants who attended the 6-month measurement sessions, we found no statistically significant differences between these groups on important baseline characteristics including age, gender, education, marital status, BMI, self-rated health, fruit/vegetable consumption, total healthful eating and average steps/day (data not shown).

Only the intervention group continued past 6 months. Between 6 and 12 months (*Minimally-Supported Phase I)*, an additional 7 individuals within the intervention group withdrew from the study and between 12 and 18 months (*Minimally-Supported Phase II)*, 2 more individuals withdrew from the study. In summary, trial retention was 76% at 6 months (i.e., 75% of participants in the intervention group and 78% of participants in the wait-list control group remained in the study). Within the intervention group, study retention decreased to 63% by 12 months and 59% by the 18-month follow-up.

### Adherence to the Health*e*Steps™ program (active phase)

When considering all individuals who were allocated to the intervention group (*n* = 59), compliance to the in-person component of the intervention was as follows: 5% attended no sessions; 17% attended one session; 10% attended two sessions; 20% attended three sessions; and 48% attended all four sessions. Across all sites, 40 participants (68%) allocated to the intervention group were classified as program completers. When considering only program completers, 30% of these participants attended 3 in-person sessions and the remaining 70% attended all 4 sessions. All participants, regardless of ‘program completer’ classification, were invited and encouraged to attend all measurement sessions.

### Baseline characteristics

As expected, due to randomization, groups were well-balanced at baseline on socio-demographic and health status characteristics (Table 1). Across both groups, most participants (98%) identified their ethnicity as white, just over three-quarters were female, and on average, participants were 58 years of age. The majority of participants were married or living as common-law (60%) and had achieved a level of education that was greater than high school (70%). Regarding health status at baseline, few participants smoked (8%); in fact, the proportion of those who reported currently smoking was lower than the proportion of Canadians who currently smoke (16.9%) [38]. About 30% of participants self-reported having high BP, high cholesterol, high blood sugar *or* type 2 diabetes, depression *or* anxiety, or back problems. A slightly higher percentage (36%) reported having arthritis or joint problems and only a small percentage (6%) reported any respiratory conditions.

**Table 1:** Baseline Participant Characteristics: Socio-demographics and Health Status

Characteristic	Comparator (*n* = 59)	Intervention (*n* = 59)
Age, y, mean (SD)	58.6 (14.7)	56.8 (12.3)
Female sex, No. (%)	48 (81.4%)	45 (76.3%)
White ethnicity, No. (%)	58 (98.3%)	57 (96.6%)
Marital status, No. (%)		
Never married	4 (6.8%)	6 (10.2%)
Married/Common-law	33 (55.9%)	38 (64.4%)
Separated/Divorced	13 (22.0%)	10 (17.0%)
Widowed	9 (15.3%)	5 (8.5%)
Education, No. (%)		
High school or less	19 (32.2%)	16 (27.1%)
Greater than high school	40 (67.8%)	43 (72.9%)
Current smoker, No. (%)	5 (8.5%)	4 (6.8%)
Current medical conditions, No. (%)		
High blood pressure	19 (32.2%)	14 (23.7%)
High cholesterol	16 (27.1%)	17 (28.8%)
Type 2 diabetes	8 (13.6%)	10 (17.0%)
High blood sugar	9 (15.3%)	10 (17.0%)
Depression or anxiety	16 (27.1%)	18 (30.5%)
Back problems	20 (33.9%)	16 (27.1%)
Arthritis/joint problems	22 (37.3%)	20 (34.5%)
Asthma, emphysema or bronchitis	5 (8.5%)	2 (3.4%)

Abbreviation: *SD* standard deviation

Baseline values of our primary outcome (average steps/day) and all secondary outcomes were also examined and contrasted between intervention and comparator groups (Table 2). Overall, groups were similar in regard to baseline values of all outcome measures. When taken together, participants reported average steps/day corresponding to a low active lifestyle (i.e., considered physically inactive and not meeting moderate to vigorous physical activity recommendations [39]) and reported sitting on average for 6 h/day. According to BMI values, participants, on average, were classified as obese class I (BMI range from 30.0 to 34.9) [40], indicating they were at a higher risk for developing health issues compared to individuals who were not classified as having obesity [41]. Participants also had systolic B*P* values approaching the normal-high BP category [42] and self-rated their health using the EQ-VAS at 70 on a scale between 0 (worst imaginable health state) and 100 (best imaginable health state) [43].

**Table 2:** Baseline Participant Characteristics: Continuous-Level Study Outcomes

Characteristic	Comparator (*n* = 59)	Intervention (*n* = 59)
Average steps/day^a^, median (IQR)	5586 (4001)^b^	5716 (4033)^c^
Total physical activity, MET-min/week^d^, median (IQR)	1451 (2781)^e^	1188 (2376)^f^
Sitting time, min/day^d^, mean (SD)	360 (240)^c^	360 (315)
Healthful eating score^g^, mean (SD)	6.4 (2.7)	6.7 (2.6)^h^
Fatty food score^i^, mean (SD)	19.7 (5.2)^h^	21.1 (6.2)
Self-rated health^i^, median (IQR)	69 (29)	70 (30)
Weight, kg, mean (SD)	86.1 (23.6)	84.2 (20.6)^h^
Body mass index, kg/m^2^, median (IQR)	30.9 (7.3)	32.0 (9.3)^h^
Waist circumference, cm, mean (SD)	102.8 (15.7)^j^	103.4 (17.1)^k^
Systolic BP, mmHg, median (IQR)	131.0 (27.0)^c^	128.5 (26.0)^b^
Diastolic BP, mmHg, median (IQR)	77.0 (15.0)^c^	76.0 (10.0)^c^

*Note:* Percentages were calculated excluding missing values

Abbreviations: *BP* Blood Pressure, *IQR* Interquartile Range, *MET* Metabolic Equivalent, *SD* Standard Deviation

^a^Measured over a 7-day period using Yamax Digiwalker (SW-200) pedometers

^b^*n* = 3 missing

^c^*n* = 2 missing

^d^From the International Physical Activity Questionnaire – Short Form

^e^*n* = 9 missing

^f^*n* = 4 missing

^g^From Starting the Conversation (lower score = more healthful eating; score range: 0–16)

^h^*n* = 1 missing

^i^From EuroQol questionnaire – visual analogue scale (higher = better state of health; score range: 0–100)

^j^*n* = 10 missing

^k^*n* = 8 missing

### Primary outcome: average steps/day

**Fig. 2:**
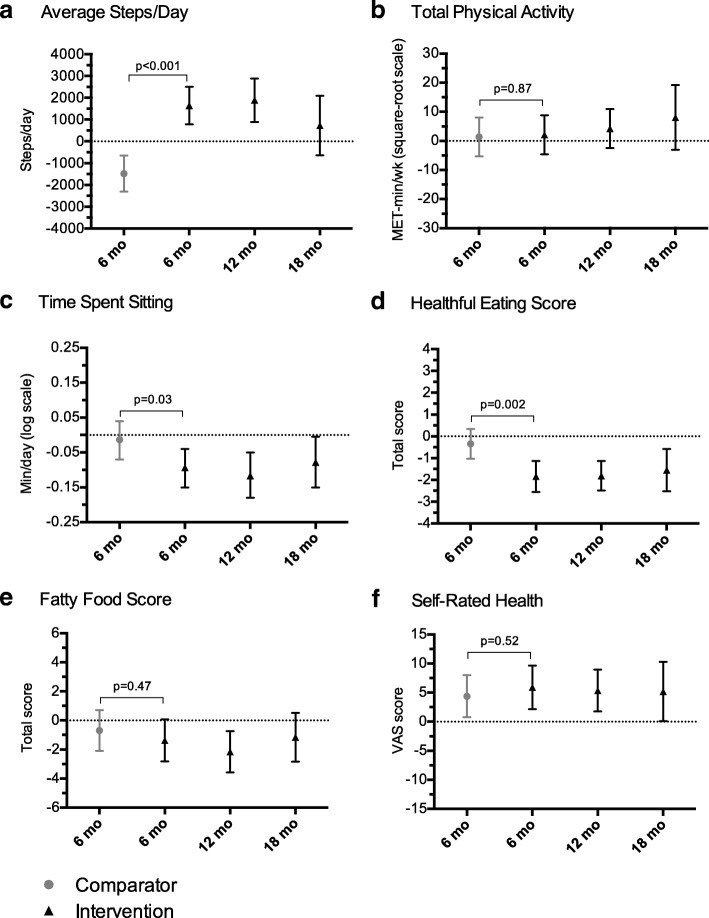
Within group mean changes from baseline in physical activity, diet, and health-related quality of life. Results displayed are estimated mean changes from baseline for primary (steps/day) and secondary outcomes focused on physical activity, diet, and self-rated health. Solid circles (Comparator) and triangles (Intervention) represent estimated group mean change from baseline and bars represent associated 95% confidence intervals. Confidence intervals not including zero (i.e., not crossing the horizontal dotted line) indicate significant differences from baseline. *P*-values correspond to between-group differences at 6 months. Panel **a** displays average steps/day measured over a 7-day period using Yamax Digiwalker (SW-200) pedometers; Panel **b** displays total physical activity in MET-minutes/week (with a square-root transformation applied) and Panel **c** displays time spent sitting on a week day in minutes/day (with a log transformation applied) both derived from the IPAQ-SF; Panel **d** displays total healthful eating calculated from the STC questionnaire (lower score = more healthful eating; score range: 0–16); Panel **e** displays total fatty food derived from a modified version of the Dietary Instrument for Nutrition Education (lower score = less fat consumption; score range: 8–68); Panel **f** displays self-rated health, which is the VAS score from the EuroQol questionnaire (higher = better state of health; score range: 0–100). *Abbreviations*: IPAQ-SF = International Physical Activity Questionnaire – Short Form; MET = Metabolic Equivalent; STC = Starting the Conversation; VAS = Visual Analogue Scale

Following the active phase of the Health*e*Steps™ program, the intervention group increased their daily physical activity, on average, by 3132 steps/day more than the comparator group (Table 3). This difference was a result of the increase observed within the intervention group (by 1646 steps/day), as well as the decrease observed within the comparator (by 1485 steps/day) after 6 months (Fig. 2a; Additional file 2: Table S2). This increase in daily steps within the intervention group remained 12 months following baseline but was no longer evident by 18 months (Fig. 2a; Additional file 2: Table S2).

**Table 3:** Differences Between Groups in Continuous-Level Study Outcomes at 6 Months (*n* = 118)

	Difference Between Groups in Mean Change at 6 Months^a^		
	Mean	95% CI	*P* value
Average steps/day	3132	1969 to 4294	< 0.001
Total physical activity, MET-min/week^b^	0.76	−8.22 to 9.74	0.87
Sitting time, min/day^c^	−0.08	−0.16 to − 0.006	0.03
Healthful eating score^d^	−1.50	−2.42 to −0.58	0.002
Fatty food score^e^	−0.68	−2.55 to 1.19	0.47
Self-rated health^f^	1.55	−3.25 to 6.35	0.52
Weight, kg	−0.46	−2.35 to 1.42	0.63
Body mass index, kg/m^2^	−0.23	−0.89 to 0.42	0.48
Waist circumference, cm	−1.53	−3.74 to 0.69	0.17
Systolic BP, mmHg	0.23	−5.02 to 5.48	0.93
Diastolic BP, mmHg	0.27	−3.26 to 3.81	0.88

Abbreviations: *BP* Blood Pressure, *CI* Confidence Interval, *MET* Metabolic Equivalents

^a^Calculated from linear mixed effects regression models that included terms for time, group x time, age, sex, site. Results should be interpreted for the intervention group (vs. comparator) at 6 months (vs. baseline)

^b^From the International Physical Activity Questionnaire – Short Form; square-root transformation applied

^c^From the International Physical Activity Questionnaire – Short Form; log transformation applied

^d^From Starting the Conversation questionnaire (lower score = more healthful eating; score range: 0–16)

^e^From a modified version of the Dietary Instrument for Nutrition Education (lower score = lower/less fat consumption; score range: 8–68)

^f^From EuroQol questionnaire – visual analogue scale (higher = better state of health; score range: 0–100)

### Secondary outcomes

#### Self-reported physical activity and sedentary time

When total physical activity was measured using the IPAQ, there were no differences between or within groups. For sedentary time, however, the intervention group decreased their time spent sitting on a weekday to a greater extent than the comparator after 6 months (Table 3) and maintained this decreased sitting time both 12 and 18 months later (Fig. 2b and c; Additional file 2: Table S2).

#### Self-reported eating

By 6 months, the intervention group decreased their healthful eating score, on average, by 1.5 points (indicating improved overall healthful eating) more than the comparator group (Table 3) and maintained improved healthful eating 12 and 18 months later (Fig. 2d; Additional file 2: Table S2). In regard to fatty food consumption, there were no observed differences between groups at 6 months (Table 3). Despite this lack of difference between groups and no change within the intervention group by 6 months, improvement (i.e., decreased consumption of fatty food) was observed within the intervention group by 12 months (Fig. 2e; Additional file 2: Table S2).

When examining sugary food consumption (<once/day vs. ≥ once/day), there were no differences between groups at 6 months or within either group at any of the follow-up time points (Table 4). For fruit and vegetable consumption, although there was no difference between groups after 6 months, favourable changes were seen within the intervention group both 6 and 12 months later (Table 4). Specifically, participants in the intervention group were: a) 1.7 times more likely to consume fruit and vegetables at least three times per day by 6 months, compared to baseline (Risk ratio: 1.73; 95% CI: 1.01 to 2.95); and b) 1.8 times more likely to consume fruit and vegetables at least three times per day by 12 months, compared to baseline (Risk ratio: 1.83; 95% CI: 1.06 to 3.17).

**Table 4:** Within Group Changes (from Baseline) in Proportions of Participants Consuming Fruit and Vegetables ≥3 times/day or Sugary Foods <once/day^a^

	Baseline		6 months			12 months			18 months		
	N	n (%)	N	n (%)	*P* ^b^	N	n (%)	*P* ^c^	N	n (%)	*P* ^c^
Fruit and vegetable consumption ≥3 times/day											
Intervention	59	25 (42.4%)	42	31 (73.8%)	0.046	36	28 (77.8%)	0.03	33	23 (69.7%)	0.09
Comparator	59	34 (57.6%)	46	27 (58.7%)	0.95	–	–	–	–	–	–
Diff. between groups					0.16						
Sugary food consumption <once/day											
Intervention	58	22 (37.9%)	42	22 (52.4%)	0.27	36	22 (61.1%)	0.13	33	18 (54.6%)	0.27
Comparator	59	24 (40.7%)	46	17 (37.0%)	0.76	–	–	–	–	–	–
Diff. between groups					0.32						

*Note:* Percentages were calculated excluding missing values

^a^Secondary categorical-level outcomes from the modified Dietary Intake Nutrition Examination. Fruit and vegetable consumption was estimated from one question and sugary food consumption was derived from a sugary food score that counted consumption of chocolates/sweets, cookies, and sugary drinks

^b^From log-linear (modified Poisson) mixed effects regression models that included terms for group, time, group x time, age, sex and site. *P*-value is for the likelihood of achieving outcome at 6 months (vs baseline)

^c^From log-linear (modified Poisson) mixed effects regression models that included terms for time, age, sex and site. *P*-value is for the likelihood of achieving outcome at 12 months or 18 months (vs baseline)

#### Health-related quality of life

Both groups improved their current self-rated health after 6 months and as a result, there was no difference detected between groups after 6 months (Table 3). On average, the intervention group improved their EQ-VAS score by 6 points while the comparator group improved their EQ-VAS score by 4 points. Within the intervention group, improved self-rated health was maintained both 12 and 18 months later (Fig. 2f; Additional file 2: Table S2).

#### Weight and body composition measures

**Fig. 3:**
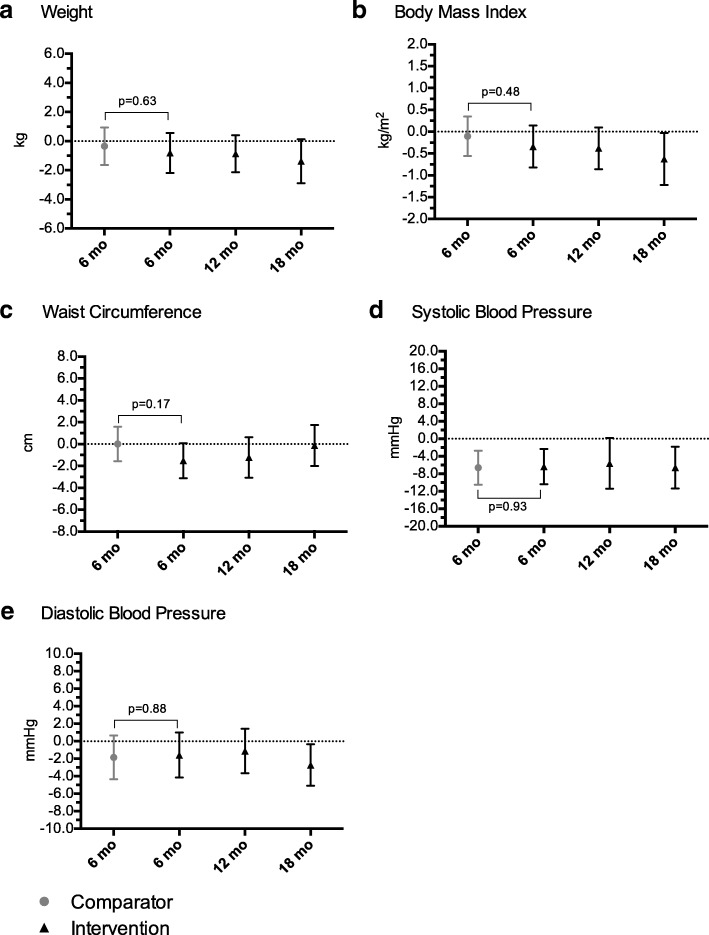
Within group mean changes from baseline in weight, body composition and cardiometabolic health. Results displayed are estimated mean changes from baseline for secondary outcomes focused on weight (Panel **a**), body composition (Panels **b** & **c**) and cardiometabolic health (Panels **d** & **e**). Solid circles (Comparator) and triangles (Intervention) represent estimated group mean change from baseline and bars represent associated 95% confidence intervals. Confidence intervals not including zero (i.e., not crossing the horizontal dotted line) indicate significant differences from baseline. *P*-values correspond to between-group differences at 6 months

There were no differences between groups at 6 months for weight, BMI or waist circumference (Table 3). For weight and waist circumference, there were also no within-group changes at any of the time points (Fig. 3a and c; Additional file 2: Table S2). Within the intervention group, while there was no change from baseline to 6 or 12 months, BMI decreased on average by 0.6 kg/m^2^ after 18 months (Fig. 3b; Additional file 2: Table S2).

#### Cardiometabolic measures

There was no difference between groups at 6 months for diastolic BP (Table 3). Similar to BMI, while there was no change within the intervention group from baseline to 6 or 12 months, diastolic BP decreased on average by 2.7 mmHg within the intervention group, after 18 months (Fig. 3e; Additional file 2: Table S2). Systolic BP decreased in both groups after 6 months and as a result, no difference was detected between the groups (Table 3). Both groups decreased their systolic BP by 6.5 mmHg on average by 6 months; the intervention group maintained this decrease to 18 months (Fig. 3d; Additional file 2: Table S2).

### Adverse events

There were no serious adverse events reported during the study period. Over the first 6 months of the study (i.e., Health*e*Steps™ program for the intervention group or the usual care/control period for the comparator group), the number of adverse events reported was numerically higher for the intervention group (18) than the comparator group (2). When only considering those adverse events that were probably or definitely related to the study/intervention, the numbers decreased to 4 in the intervention group and 0 in the comparator group. Of the 4 adverse events within the intervention group, all were musculoskeletal in nature [i.e., back pain (2), foot pain (1), and plantar fasciitis (1)] and events that would be anticipated from a lifestyle program designed to increase physical activity in individuals at increased risk for chronic disease.

## Discussion

With a very large proportion of Canadians living with one or more modifiable risk factors for chronic disease [2] and considering the rise in the costs associated with health care [4], lifestyle programs are essential to primarily prevent, but also assist with treatment and management of chronic disease burden [5]. Our results suggest that individuals who participated in the Health*e*Steps™ lifestyle prescription program increased their physical activity (i.e., step counts/day), decreased their sitting time, and improved their overall healthful eating to a greater extent than individuals who continued with their usual activities after 6 months in the program. Furthermore, exploratory analyses showed that these individuals maintained these outcomes 12 months later (within-group changes), following a minimally-supported phase that involved only online technology and telephone supports. As well, these exploratory results indicated that these individuals further maintained their decreased sedentary time and improved healthful eating after 18 months, when only publicly available technology resources were encouraged. Therefore, the results of this trial suggest that Health*e*Steps™ can effectively promote a healthy lifestyle and lead to positive health behaviour changes.

Research interventions promoting physical activity and healthful eating have shown unequivocal results in reducing chronic disease risk in controlled settings [44–47], although it is noteworthy that effective methods for implementing this evidence into everyday primary care practice warrants deeper investigation. In this context, evidence from the Health*e*Steps™ lifestyle prescription program supporting positive lifestyle changes is encouraging given the pragmatic nature of our study. We were able to demonstrate that changes observed in physical activity, sitting time and healthful eating are achievable without the need for special equipment and arduous staff training. Moreover, in our approach, we trained staff at their site to provide the intervention to the participants; we also made free technology available and were careful that the study intervention would not disrupt practice or add to the burden of the clinical staff. Also, positive changes in sedentary time and healthful eating within the intervention group were maintained 18 months after participants continued in the program via remote monitoring only (e.g., via Health*e*Steps™ Smartphone app) and with less impact on practice staff, showing promise for long-term sustainability. This paper follows our process evaluation manuscript which details the acceptability of the program from participant and coach perspectives [48].

Despite positive changes described above, the Health*e*Steps™ lifestyle prescription program did not lead to significant changes in weight and body composition (BMI and waist circumference) or cardiometabolic measurements (SBP and DBP; although DBP decreased by 18 months). As a possible explanation to these findings, we speculate that the components of our intervention program might not have been intense enough to elicit significant changes in these outcomes compared to other similar protocols in previous literature [49–51]. Furthermore, it is plausible that changes in weight and body composition could have been hindered due to individual variability in response to the program; the fact that we did not specifically recruit obese or hypertensive participants; as well as our focus on improving healthy eating behaviours but not controlling/reducing calorie intake [52–54]. In addition, it is relevant to note that the lack of changes in other lifestyle risk factors reported in this study might reflect the increased variability associated with implementing a pragmatic design.

Finally, although participants demonstrated changes in steps/day, which is an objective measure of physical activity, we did not observe changes in IPAQ scores for participants. A recent report has shown that IPAQ data are less precise in capturing change over time in walking compared to step counts [55]. Even though IPAQ is a valid method of measuring self-reported physical activity [26], the self-reported nature of the measure could impart imprecision in the outcome measured and account for the lack change in our study [55].

### Limitations and future directions

One of the major shortcomings of this study was a retention rate lower than was expected—despite home visits being completed by the research team. Only two thirds of participants completed the program, regardless of the fact that repeated attempts were made by the research team to contact participants. Consequently, this leads us to believe that at higher completion rates, stronger treatment effects could have been observed. Additionally, we did not conduct assessments with the wait list group at 12 and 18 months, which would have led to more robust data and the ability to examine changes between intervention and comparator at these timepoints; the long-term results should be interpreted with caution and these analyses should be considered exploratory.

Although we made the Health*e*Steps™ app available at no cost to participants in Android and iOS systems, smartphone technology was required and may not have been accessible to some participants. Further, because of the multicomponent nature of the intervention, it is difficult to determine whether behavioural changes over the course of the first 6 months were due to counseling or technology supports available or both. Our primary and secondary outcomes (besides weight, BMI, waist circumference and blood pressure), were self-reported by participants; this should be taken into consideration when reviewing and interpreting the results [56]. Lastly, there may have been variations in the results at 12 and 18 months due to the timing of the measurements occurring in the winter (12-month assessments) and summer (18-month assessments). As identified in previous publications about the Health*e*Steps™ program, winter weather was identified as a barrier to physical activity [48].

Previous research suggests that self-monitoring and goal setting, along with counselling, have shown greater success in leading to the maintenance of behaviour changes in the long term within individuals at risk for chronic disease [57]. This current study indicates that Health*e*Steps™ has the potential to positively impact individuals’ long-term maintenance of behaviour change; however, further research is necessary to more fully explore these effects including follow-up with a wait-list comparison group at 12 and 18 months, and a longer follow-up period past 18 months.

New approaches for the implementation and the sustainability of lifestyle programs are needed to attenuate the burden of chronic disease in the future. Indeed, attention should be given to innovative strategies to enhance participant adherence and retention in programs, considering retention rates in our study. A recent study has reported the success of incentivizing lifestyle programs to improve adoption and retention in community programs in the UK, USA and South Africa [58].

Additionally, more sophisticated strategies to enhance healthful eating while reducing calorie intake and promoting changes to body composition are needed, which might also benefit arterial blood pressure regulation and overall cardiometabolic health, thereby reducing the risk for chronic disease in this population. Finally, integration of in-person and remote delivery of healthy lifestyles appears feasible in rural and urban settings and may even provide participants with options that appeal to their needs. These considerations could inform and enable future research aimed at examining the *true effects* of initiatives such as Health*e*Steps™ in people living in rural and remote communities.

## Conclusions

Results from this study suggest that the Health*e*Steps™ lifestyle prescription program is effective at increasing physical activity, decreasing weekday sitting time, and improving healthful eating in adults at increased risk for chronic disease after 6 months. Furthermore, results suggest that these individuals can maintain these behaviours 12 months later and, in some instances, even 18 months later, with minimal support, showing promise for long-term sustainability. Further study is required to improve retention and adherence using pragmatic methods.

## Additional files

Additional file 1: **Table S1.** Sample of Step Count Guide for Participants. Physical activity (step count) prescription form. (DOCX 20 kb)

Additional file 2: **Table S2.** Within Group Mean Changes from Baseline in Continuous-Level Study Outcomes. Supplementary results. (DOCX 25 kb)

## Data Availability

The datasets used and/or analysed during the current study are available from the corresponding author upon request.

## Abbreviations

BMI: Body Mass Index
BP: Blood Pressure
DBP: Diastolic Blood Pressure
DINE: Dietary Instrument for Nutrition Education
EQ-VAS: EuroQol Visual Analog Scale
IPAQ: International Physical Activity Questionnaire
MET: Metabolic Equivalent
PAR-Q: Physical Activity Readiness Questionnaire
SBP: Systolic Blood Pressure
SMART: Specific, Measurable, Attainable, Realistic, and Timely
STEP: Step Test and Exercise Prescription
