# Supplementary material for: The HealtheSteps™ lifestyle prescription program to improve physical activity and modifiable risk factors for chronic disease: a pragmatic randomized controlled trial
Source: BMC Public Health. 2019 Jun 28;19:841. doi: 10.1186/s12889-019-7141-2 (PMC6599363; doi:10.1186/s12889-019-7141-2)
Supplement: Supplementary file 1 — Table S1. Sample of Step Count Guide for Participants. Physical activity (step count) prescription form. (DOCX 20 kb) [file 12889_2019_7141_MOESM1_ESM.docx]

**Table S1. Sample of Step Count Guide for Participants**

**Physical Activity (Step Count) – Prescription (Rx)**

Participant ID#: HESP

Date Completed (dd/mm/yy):

/ /

*It is recommended that we take approximately 10,000 steps per day*

1. **Identifying the Number of Steps You Should Take Each Day (Physical Activity Rx)**

Baseline Daily Step Count (Average):

**Each week, try to *increase* your daily step count using the information provided in the table below.**

| **Circle** | **Increase Baseline Step Count By…** |
| --- | --- |
| **Week 1** | Baseline Step Count + 1,500 steps on at least 3 days of the week |
| **Week 2** | Baseline Step Count + 1,500 steps on at least 3 days of the week |
| **Week 3** | Baseline Step Count + 1,500 steps on at least 3 days of the week |
| **Week 4** | Baseline Step Count + 1,500 steps on at least 5 days of the week |
| **Week 5** | Baseline Step Count + 1,500 steps on at least 5 days of the week |
| **Week 6** | Baseline Step Count + 3,000 steps on at least 3 days of the week |
| **Week 7** | Baseline Step Count + 3,000 steps on at least 3 days of the week |
| **Week 8** | Baseline Step Count + 3,000 steps on at least 5 days of the week |
| **Week 9** | Baseline Step Count + 3,000 steps on at least 5 days of the week |
| **Week 10** | Baseline Step Count + 4,500 steps on at least 3 days of the week |
| **Week 11** | Baseline Step Count + 4,500 steps on at least 3 days of the week |
| **Week 12** | Baseline Step Count + 4,500 steps on at least 5 days of the week |

A SAMPLE Physical Activity Rx

- - **Baseline Daily Step Count = 3300 steps**

*Week 1 - Physical Activity Rx:*

= BASELINE {3300 steps} + 1500 STEPS on 3 days of the week

= 4800 daily steps on at least 3 days of the week and 3300 steps on the other days of the week
