# Supplementary material for: The HealtheSteps™ lifestyle prescription program to improve physical activity and modifiable risk factors for chronic disease: a pragmatic randomized controlled trial
Source: BMC Public Health. 2019 Jun 28;19:841. doi: 10.1186/s12889-019-7141-2 (PMC6599363; doi:10.1186/s12889-019-7141-2)
Supplement: Supplementary file 2 — Table S2. Within Group Mean Changes from Baseline in Continuous-Level Study Outcomes. Supplementary results. (DOCX 25 kb) [file 12889_2019_7141_MOESM2_ESM.docx]

**Table S2. Within Group Mean Changes from Baseline in Continuous-Level Study Outcomes**

|  | **Comparator (n=59)** |  | **Intervention (n=59)** | | |
| --- | --- | --- | --- | --- | --- |
|  | **Change at 6 mo.**  **Mean (95% CI)^a^** |  | **Change at 6 mo.**  **Mean (95% CI)^a^** | **Change at 12 mo.**  **Mean (95% CI)^b^** | **Change at 18 mo.**  **Mean (95% CI)^b^** |
| Average steps/day | -1485  (-2312 to -659) |  | 1646  (786 to 2507) | 1890  (888 to 2892) | 728  (-635 to 2091) |
| Total PA, MET-min/wk^c^ | 1.37 (-5.32 to 8.06) |  | 2.13 (-4.59 to 8.85) | 4.25 (-2.45 to 10.94) | 8.06 (-3.08 to 19.19) |
| Sitting time, min/d^d^ | -0.01 (-0.07 to 0.04) |  | -0.09 (-0.15 to -0.04) | -0.12 (-0.18 to -0.05) | -0.08 (-0.15 to -0.005) |
| Healthful eating score^e^ | -0.35 (-1.03 to 0.34) |  | -1.84 (-2.56 to -1.13) | -1.81 (-2.49 to -1.13) | -1.55 (-2.52 to -0.58) |
| Fatty food score^f^ | -0.70 (-2.10 to 0.71) |  | -1.38 (-2.83 to 0.08) | -2.16 (-3.58 to -0.75) | -1.16 (-2.84 to 0.52) |
| Self-rated health^g^ | 4.37 (0.76 to 7.98) |  | 5.92 (2.17 to 9.67) | 5.36 (1.79 to 8.93) | 5.19 (0.08 to 10.30) |
| Weight, kg | -0.35 (-1.64 to 0.94) |  | -0.81 (-2.18 to 0.56) | -0.86 (-2.13 to 0.41) | -1.37 (-2.88 to 0.13) |
| Body mass index, kg/m^2^ | -0.10 (-0.56 to 0.35) |  | -0.34 (-0.82 to 0.14) | -0.38 (-0.86 to 0.10) | -0.63 (-1.22 to -0.03) |
| Waist circumference, cm | 0.01 (-1.57 to 1.58) |  | -1.52 (-3.12 to 0.08) | -1.22 (-3.07 to 0.62) | -0.12 (-2.00 to 1.75) |
| Systolic BP, mmHg | -6.61 (-10.52 to -2.70) |  | -6.38 (-10.43 to -2.33) | -5.64 (-11.46 to 0.18) | -6.58 (-11.35 to -1.81) |
| Diastolic BP, mmHg | -1.84 (-4.35 to 0.67) |  | -1.57 (-4.14 to 1.00) | -1.10 (-3.65 to 1.44) | -2.71 (-5.08 to -0.34) |
| **Abbreviations:** BP = Blood Pressure; CI = Confidence Interval; MET = Metabolic Equivalents; PA = Physical Activity  ^a^Calculated from linear mixed effects regression models that included terms for time, group x time, age, sex, site. Results should be interpreted at 6 months (vs. baseline).  ^b^Calculated from linear mixed effects regression models that included terms for time, age, sex, site (included Health*e*Steps group only). Results should be interpreted at 12 months (vs. baseline) or 18 months (vs. baseline).  ^c^From the International Physical Activity Questionnaire – Short Form; square-root transformation applied.  ^d^From the International Physical Activity Questionnaire – Short Form; log transformation applied.  ^e^From Starting the Conversation questionnaire (lower score = more healthful eating; score range: 0-16).  ^f^From a modified version of the Dietary Instrument for Nutrition Education (lower score = lower/less fat consumption; score range: 8-68).  ^g^From EuroQol questionnaire – visual analogue scale (higher = better state of health; score range: 0-100). | | | | | |
